# Localized tetanus bacillus infection following open metatarsal fracture in an adult: A case report

**DOI:** 10.1097/MD.0000000000042633

**Published:** 2025-05-23

**Authors:** Xiaoqiang Wang, Xingzhang Yao, Jun Zhao, Xingwen Xie, Xingsheng Wang, Shaobo Jing, Kai Li, Zhaoyang Jiang, Sixuan Chen, Shihong Xu

**Affiliations:** a Gansu Provincial Hospital of Traditional Chinese Medicine, Gansu, China; b Affiliated Hospital of Gansu University of Traditional Chinese Medicine, Gansu, China.

**Keywords:** closed negative pressure drainage of wounds not suitable for anaerobes, early bacteriological culture of fresh wounds, localized tetanus bacillus infection in adults, thorough debridement

## Abstract

**Rationale:**

Localized tetanus is a rare subtype characterized by muscle spasms in the vicinity of necrotic wounds and is often misdiagnosed due to overlapping symptoms. Tetanus has a high mortality rate and is tricky to diagnose and treat clinically. Our case report is about a 61-year-old male patient who was admitted to a local hospital with an open fracture of the right foot and underwent emergency debridement of the open fracture site, internal fixation, and closed negative pressure drainage of the wound, and developed a localized *Mycobacterium tetani* infection in the postoperative period. We discussed the diagnosis and treatment process of this adult open metatarsal fracture combined with localized tetanus bacillus infection, and put forward the deficiencies in the diagnosis and treatment process of the disease and treatment suggestions, aiming to provide a reference for the clinical diagnosis and treatment of this disease.

**Patient concerns:**

The outcome of the treatment is the most important aspect of the patient’s concern. The patient was treated, the tetanus bacillus infection was controlled, involuntary convulsions disappeared, and the patient’s pain was reduced. Follow-up skin healing was good and the patient resumed normal activities, reducing the economic burden.

**Diagnoses:**

The patient had an open fracture caused by trauma, and after surgical treatment, he developed paroxysmal right knee flexion contracture without any obvious causative factors, and the right triceps brachii muscle of the lower leg was twitching, spasmodic, stiff, and painful. After admission, through clinical symptoms, combined with bacteriological culture, the final diagnosis was localized type tetanus bacillus infection after an open metatarsal fracture in adults.

**Interventions:**

After admission, the patient was treated with a combination of thorough debridement, internal fixation with Kirschner needle, closed negative pressure drainage of the wound, neural tissue anesthesia, intramuscular injection of tetanus antitoxin, ceftriaxone combined with metronidazole, and vancomycin bone cement.

**Outcomes:**

The patient’s condition improved, and the skin healed well on follow-up.

**Lessons:**

From this case, we conclude that the elderly are not actively immunized against tetanus and tetanus vaccination is recommended for those over 65 years of age. Open wound patients first seen at an outside hospital to ensure and perform passive immunization. In the management of open wounds, preoperative bacteriological culture of fresh wounds in the emergency department is essential to guide therapeutic use of medications to prevent the development of infection and target anti-infective therapy. Early and thorough debridement of open wounds is effective in removing residual tetanus bacilli and their spores from the wound and reducing the release of toxins. Ineffective nerve block anesthesia can be used as a reference indicator of tetanus bacillus infection to aid diagnosis and treatment. Closed negative pressure drainage of the wound promotes the propagation of anaerobic bacteria, and the use of closed negative pressure drainage of the wound is avoided when the bacteria suspected of infection are anaerobic bacteria. Vancomycin bone cement has many advantages, such as long duration of action and small side effects, which can effectively control the infection.

## 1. Introduction

Tetanus infection is an acute specific disease characterized by persistent tonic contractions and paroxysmal spasms of skeletal muscles throughout the body. Tetanus bacilli and their spores are widely present in soil, animals, and human feces. After a traumatic injury resulting in an open fracture in a patient, tetanus bacillus enters the injury site through the open wound and rapidly grows and multiplies, and produces exotoxin, which leads to tetanus infection. The clinical triad of typical tetanus bacillus infection is muscle tonus, spasticity, and autonomic dysfunction.^[1]^ Tetanus has an almost 100% case-fatality rate in the elderly and infants without medical intervention, and even after comprehensive ICU treatment, the case-fatality rate is still as high as 30% to 50%.^[2]^ In contrast, localized tetanus, which is uncommon, is a condition in which the toxin affects only the muscles in the vicinity of the site of infection, resulting in limited muscle stiffness and spasm, characterized by persistent contracture of the muscles at the site of injury.

## 2. Case reports

### 2.1. Patient information

**Figure 1.**
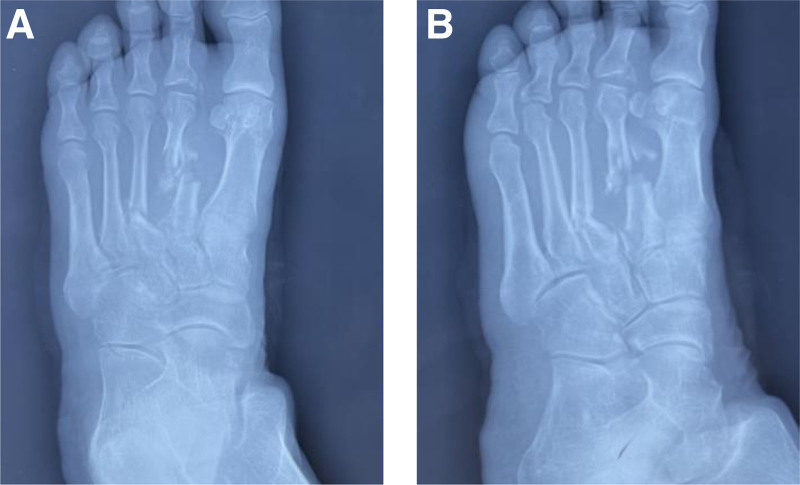
Fractures due to trauma. (A) X-ray: right foot orthotopic. (B) X-ray: right foot oblique position.

A 61-year-old male patient, Han ethnicity, farmer, was admitted to the hospital with “localized high skin temperature in the distal part of the right calf, paroxysmal muscle twitching, spasm and stiffness for more than 3 days.” Open fracture of the right foot due to trauma 10 days prior to admission. There was no history of other illnesses, no history of genetic disorders or family history. The patient was admitted to a local hospital where X-rays showed multiple comminuted metatarsal fractures of the right foot (Fig. 1(A) and (B)). The patient was admitted to the hospital and underwent debridement, internal fixation with a plate and closed negative pressure suction of the trauma. On the 4th postoperative day, the wound appeared to have a bad smell, the foot was obviously red, swollen and painful, and the right calf was obviously swollen, accompanied by chills and fever, which was treated by removing the closed negative pressure suction material from the trauma, removing the infected and necrotic tissues, routine cleaning and changing of medication, and symptomatic treatment of anti-infection. Paroxysmal right knee flexion contracture, jerks, spasms, stiffness, and pain in the triceps brachii muscle of the right calf occurred on the 7th postoperative day without any obvious trigger.

### 2.2. Admission performance

**Figure 2.**
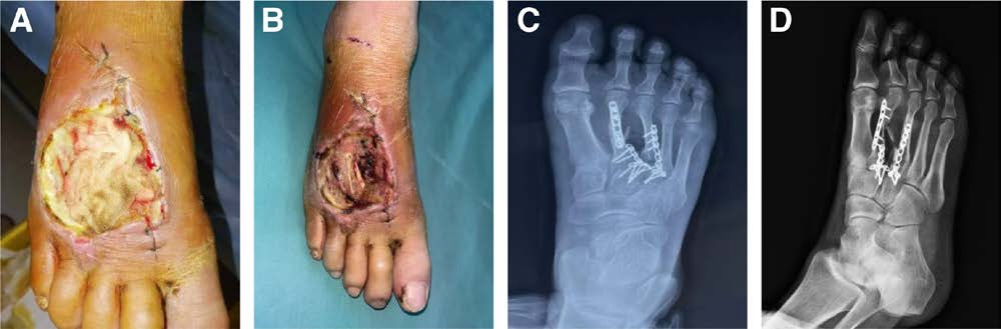
Admission photos. (A) The dorsal foot wound was gauze-filled with purulent exudate. (B) Partial necrosis of the trauma with exposed tendons, bone, and some internal fixation. (C) X-ray: orthostasis after internal fixation. (D) X-ray: oblique position after internal fixation.

Localised high skin temperature, paroxysmal muscle twitching, spasms and rigidity in the distal right calf, wound oozing purulent discharge, flocculent necrosis of soft tissues, localized skin pigmentation (Fig. 2(A)), and odor could be smelled. A soft tissue necrotic defect of approximately 6 × 4 cm was seen in the right foot, with exposed tendons and bone and yellowing of the bone (Fig. 2(B)). A wound of approximately 4 cm in length can be seen on the medial side of the right plantar foot, with slight localized erythema, a small amount of exudate and a warm skin temperature. Numbness of the dorsum and plantar aspect of the right foot with limited toe movement. X-rays showed a fracture of the proximal second metatarsal of the right foot after internal fixation of fractures of the second and third metatarsals of the right foot (Fig. 2(C) and (D)).

### 2.3. Changes in condition

On the first postoperative day, the frequency of muscle twitching and spasms in the right lower limb was reduced and the pain was significantly relieved.

On the second postoperative day, the frequency of pain in the affected limb, twitching and spasms in the calf muscles was significantly reduced and the pain was tolerable.

On the third postoperative day, the pain subsided with symptomatic treatment but recurred.

On the fourth postoperative day, the pain in the surgical area was slightly relieved, and the twitching, cramping and stiffness of the right calf was unbearable.

On the fifth postoperative day, there was a marked increase in the frequency of muscle spasm and twitching in the right calf, with marked local stiffness and poor toe movement.

On the fourteenth postoperative day, the patient’s condition was stable and there was no obvious muscle twitching, spasm or stiffness discomfort in the right lower limb.

### 2.4. Confirmation of tetanus bacillus infection

**Figure 3.**
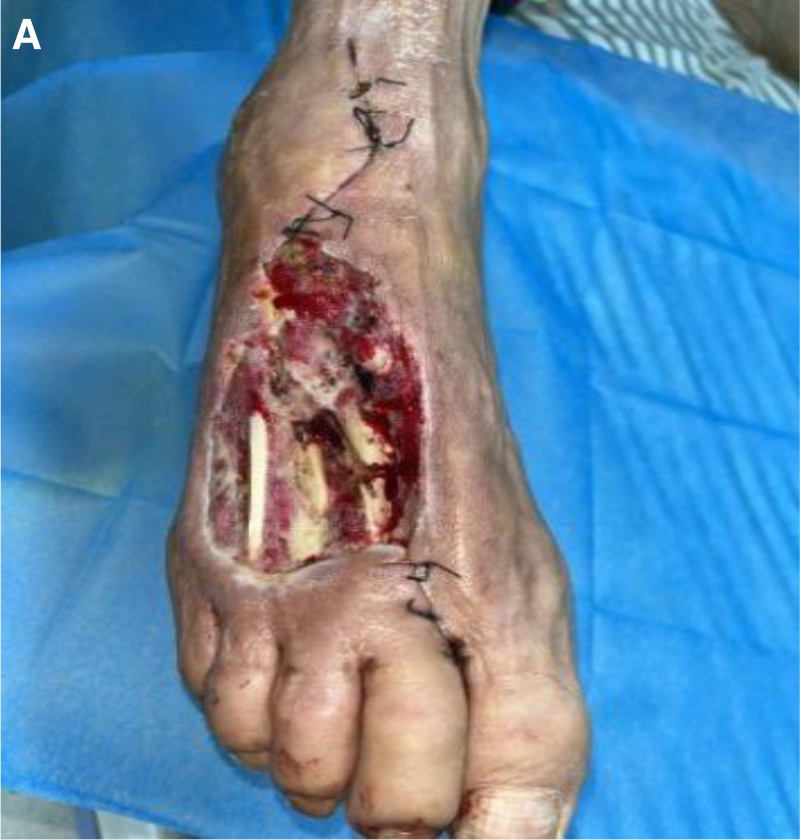
Second intraoperative picture after admission. (A) A small amount of purulent exudate on the wound, with a small amount of granulation tissue already present.

Removal of the closed negative pressure drainage material from the wound showed granulation tissue growth on the wound (Fig. 3(A)), and *Bacillus cereus* was cultured from all 4 tissues at the site of injury (Table 1), and the clinical symptoms were consistent with the features of tetanus infection. Nerve block anesthesia was given after the second operation, and the symptoms were slightly relieved but recurred, and we diagnosed localized tetanus bacillus infection after an open fracture in an adult by excluding other possible diseases based on the symptoms. Follow-up at 15 months postoperatively showed good skin healing.

**Table 1:** Tissue, secretion, and blood culture results.

Targets	Culture time	Result
General bacterial culture and identification (secretions)	November 08, 2023	Identification result: *Enterobacter cloacae* drug sensitivity result: *Enterobacter cloacae* drug
General bacterial culture and identification (secretions)	November 08, 2023	Identification result: *Enterobacter cloacae* drug sensitivity result: *Enterobacter cloacae* drug
General bacterial culture and identification (tissue)	November 09, 2023	Identification result: *Enterobacter cloacae* drug
General bacterial culture and identification (tissue)	November 10, 2023	Identification result: *Enterobacter cloacae* drug
General bacterial culture and identification (tissue)	November 11, 2023	Identification result: *Enterobacter cloacae* drug
Anaerobic bacteria culture and identification (tissue)	November 16, 2023	Identification result: *Enterobacter cloacae*, group pantobacter drug sensitivity result: *Enterobacter cloacae* drug
General bacterial culture and identification (tissue)	November 16, 2023	Identification results: *Stenotrophomonas maltophilia*, *Staphylococcus aureus* drug susceptibility results: *Stenotrophomonas maltophilia* drug
Anaerobic bacteria culture and identification (tissue)	November 16, 2023	Identification result: *Enterobacter cloacae*, group pantobacter drug sensitivity result: *Enterobacter cloacae* drug
Venous blood culture (anaerobic bottle) (Venous blood)	November 16, 2023	Identification result: aseptic growth
Venous blood culture (aerobic bottle) (Venous blood)	November 16, 2023	Identification result: aseptic growth
Aspergillus cell wall galactomannan Test (GM test)	November 17, 2023	Identification results:0.121
Brucella tiger-red plate test	November 17, 2023	Identification result: negative
General bacterial culture and identification (secretions)	November 19, 2023	Identification result: *Stenotrophomonas maltophilia* drug susceptibility result: *Stenotrophomonas maltophilia* drug
Anaerobic bacteria culture and identification (secretions)	November 19, 2023	Identification result: *Stenotrophomonas maltophilia* drug
General bacterial culture and identification (secretions)	November 30, 2023	Identification result: *Stenotrophomonas maltophilia* drug susceptibility result: *Stenotrophomonas maltophilia* drug

### 2.5. Treatment

**Figure 4.**
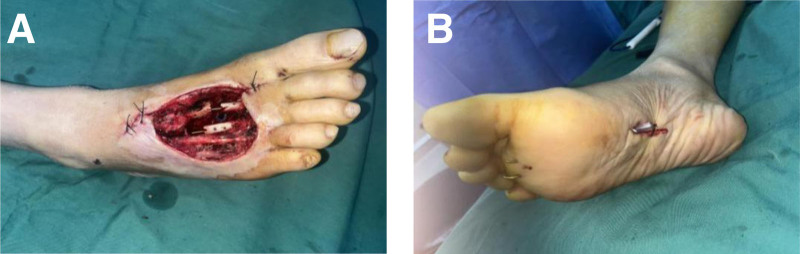
First intraoperative picture after admission. (A) Thorough debridement, removal of original internal fixation, and titanium pin refixation. (B) The wound is connected to the sole of the foot, and a drain is placed.

After admission, open fracture debridement and closed negative pressure drainage were performed under intralesional anesthesia. Intraoperatively, there was soft tissue swelling at the periphery of the trauma; subcutaneous adipose tissue degeneration and necrosis at the periphery of the trauma; purplish and leathery margin of the trauma on the right plantar surface; the plate screw was completely exposed; clay-like material adhered to the distal end of the tendon; the 2nd metatarsal stem was wire-bound; there was a comminuted fracture at the base of the 3rd metatarsal; the severed ends of the extensor tendons of the 2nd and 3rd toes had localized necrosis; and necrotic tissue was evident locally on the 4th metatarsal. Removal of all plate screws; excision of infected inactivated tissue in the trauma; excision of the 2nd and 3rd toe extensor tendons; total necrosis of the tissue around the fracture break and below the metatarsal, with a small amount of yellow pus. Cleaning below the metatarsal bone through the gap between the broken ends of the 2nd and 3rd metatarsal bones, most of the dorsal interosseous muscles, transversus adductors, and earthworms were degenerated and necrotic, the soft tissues around the distal ends of the 2nd and 3rd metatarsals were necrotic, the distal stumps of the extensor tendons of the toes were locally necrotic, the transversus short flexors of the foot were partially necrotic, and the transversus longus flexor tendon had a good continuity; the dorsal foot and the plantar trauma was completely penetrated. Two titanium pins were threaded longitudinally through the bone to fix the 2nd and 3rd diaphysis, respectively, and the 2nd and 3rd metatarsals, respectively (Fig. 4(A)), and a drainage tube was inserted from the dorsalis pedis wound into the plantar wound (Fig. 4(B)) and the wound was covered with a trauma-enclosed, negative pressure drainage device that carries a retention-irrigation channel.

On the fifth day, due to the recurrence of the disease, traumatic debridement and closed negative pressure drainage were performed again. During the operation, the size of the right dorsal foot trauma was about 10 × 6 cm, with some tendons and bone tissues exposed, and a little soft tissue necrosis on the trauma surface; the metatarsal fracture was well aligned, with a local bone defect, and granulation hyperplasia could be seen in the defect area; the dorsal and plantar trauma was through the medial side of the foot, and the tendons of long extensor tendon and long extensor tendon of the toes were obvious with local scarring, and the superficial peroneal nerve was intact, with adhesion and edema obvious, and the superficial peroneal nerve intermediate peroneal nerve was disconnected, and the dorsal foot artery was disconnected from the upper part of the trauma and ligated and embolized. The superficial peroneal artery was disconnected from the upper part of the trauma and ligated and embolized, the medial branch of the deep peroneal nerve was disconnected from the upper part of the trauma, the lateral branch of the deep peroneal nerve was intact, and the plantar tendon membrane was necrotic in a few instances.

Postoperative injection of tetanus antitoxin 6000 U intramuscularly twice daily for 4 days to neutralize the toxin, reduce symptoms and stop further disease progression. Ceftriaxone 4 g combined with metronidazole 0.5 g to treat mixed bacterial infection. Adjust the patient’s ward, prohibit the ward personnel from large movement, prohibit noise and light stimulation, draw the curtains, and keep the environment quiet. Nutritional diet.

**Figure 5.**
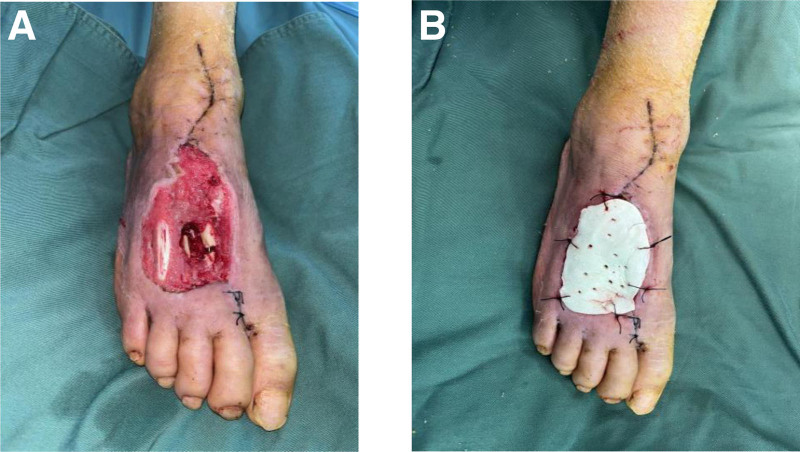
Third intraoperative picture after admission. (A) Reduced wound size, no exudate, lots of granulation tissue. (B) Vancomycin-filled, suture-fixed.

**Figure 6.**
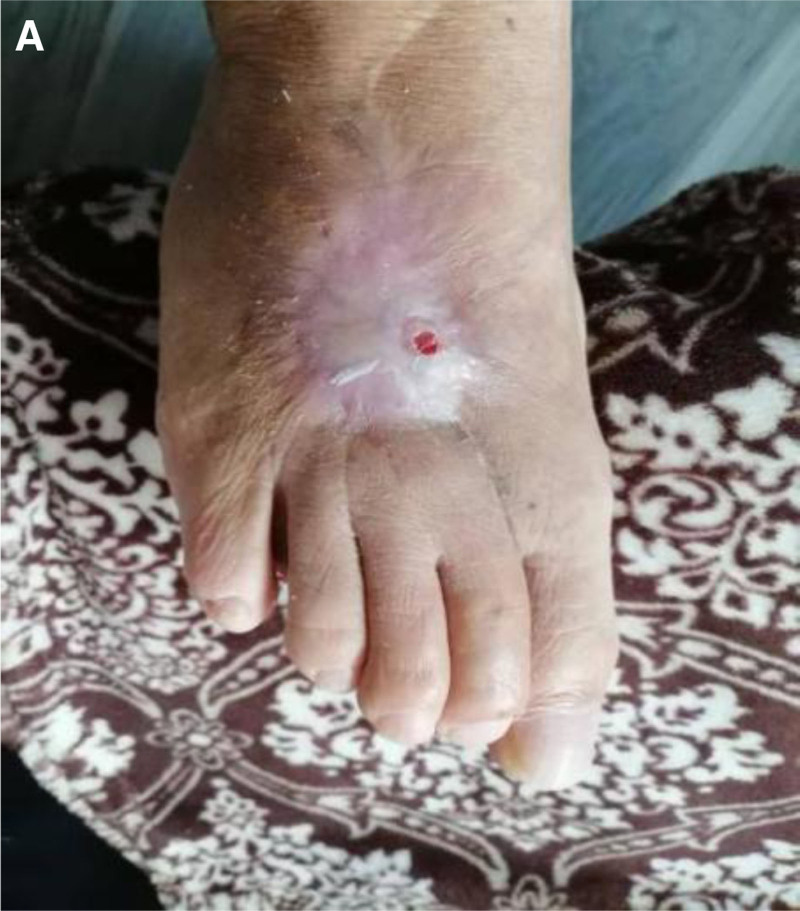
(A) Follow-up at 15 months postoperatively showed good wound healing.

On the fifteenth day, the trauma was debrided and covered with bone cement. Intraoperatively, the broken end of the right dorsal foot fracture and local bone were exposed, and most of the granulation tissue in the wound was well grown (Fig. 5(A)), and vancomycin bone cement was covered over the wound (Fig. 5(B)). Patient follow-up (Fig. 6(A)) showed good skin healing. None of the bacteriological examinations showed *Enterobacter cloacae* (Table 1). Venous blood examination was better than before (Table 2).

**Table 2:** Changes in relevant inflammatory markers.

Targets	Reference scope	November 08, 2023	November 12, 2023	November 16, 2023	November 19, 2023	November 24, 2023	December 01, 2023
Leukocyte (10^9^/L)	3.5–9.5	5.24	5.83	7.77	4.23	4.08	2.89
Neutrophil ratio (%)	40–75	83.70	74.50	79.60	62.70	59.80	58.50
Lymphocyte ratio (%)	20–50	9.40	20.20	12.40	25.30	27.50	32.20
Monocyte ratio (%)	3–10	3.20	4.80	7.50	8.70	9.30	6.60
Eosinophil ratio (%)	0.4–8	3.10	0.00	0.00	2.60	2.00	1.70
Basophilic granulocyte ratio (%)	0–1	0.40	0.20	0.10	0.50	1.20	0.70
Neutrophil absolute value (10^9^/L)	1.8–6.3	4.39	4.34	6.19	2.65	2.44	1.69
Lymphocyte absolute value (10^9^/L)	1.1–3.2	0.49	1.18	0.96	1.07	1.12	0.93
Monocyte absolute value (10^9^/L)	0.1–0.6	0.17	0.28	0.58	0.37	0.38	0.19
Eosinophils absolute value (10^9^/L)	0.02–0.52	0.16	0.00	0.00	0.11	0.08	0.05
Basophils absolute value (10^9^/L)	0–0.06	0.02	0.01	0.01	0.02	0.05	10.02
C-reactive protein (mg/L)	0–6	–	46.02	13.35	14.52	0.5	0.13
Plasma D-dimer assay (mg/L)	0–0.55	4.78	2.20	2.48	–	1.91	1.82
Erythrocyte sedimentation rate (ESR) (mm/h)	0–15	–	–	103	82	22	–
Procalcitonin (ng/L)	0–0.5	–	0.71	0.08	0.08	0.08	0.06

## 3. Discussion

### 3.1. Tetanus bacillus sources

Tetanus infection is caused by spores of the tetanus bacillus that invade the body’s tissues through broken skin and mucous membranes, develop into a proliferating organism and multiply in an oxygen-poor environment, releasing spasmodic toxins. The most common cause of tetanus infection is the puncture of a rusty nail into the foot, rare routes of tetanus infection include abrasions, surgical procedures, insect bites, and intravenous injections, however, no cases of tetanus infection due to injections have been found in 20% of tetanus patients.^[3]^ Neonatal-type tetanus is mainly caused by cutting the umbilical cord with unclean instruments or bandaging the umbilical cord stump with dressings contaminated with Mycobacterium tetani. Head-type tetanus is extremely rare and is mostly caused by head injury or otitis media.

### 3.2. Tetanus bacillus pathogenesis

After invasion of localized rupture of skin or mucous membranes by tetanus bacilli spores, they proliferate as propagules in an anaerobic microenvironment and produce tetanus spasmodic toxin to act. After entering the body, the tetanus spasmodic toxin first binds to gangliosides in the nerve endings and travels retrograde along the nerve sheaths through the spinal nerve roots into the neurons in the anterior horns of the spinal cord and then upwards to the brain stem cells. Secondly the tetanus spasmodic toxin attaches to serum globulin, is absorbed by the lymphatic fluid and reaches the central nervous system through the circulation. In addition tetanus spasmodic toxin selectively inhibits neurons and prevents the release of neurotransmitter inhibitors, which leads to a dysregulation of the systolic diastolic balance of the extensor and flexor tendons, as well as strong contractions of the extensor and flexor tendons, which manifest as localized spasms and convulsions. Finally neuromuscular receives and transmits signals through synapses, and tetanus spasmodic toxin inhibits this process, leading to the concentration of acetylcholine in the cell membrane conjugates, and with the prolongation of time and accumulation of the quantity of acetylcholine gathered in the cell membrane conjugates, frequent impulsive signals to the peripheral nerves are sent to the organism, and the organism receives the signals, and continuously enhances the muscle tone and muscle spasm of the organism.^[4]^

### 3.3. Clinical manifestations

Systemic tetanus is the most common and recognizable type, accounting for more than 80% of cases, with symptoms appearing gradually about 3 to 21 days after infection, and symptoms usually worsening within a week, with the most noticeable symptoms being painful muscle contractions, mainly in the biting and neck muscles, leading to clenching of the teeth or “jaw locking,” contraction of the facial muscles leading to frowning, and reflex spasms due to backward bending of the head, neck, and spine.^[5]^ Neonatal-type tetanus usually occurs 3 to 7 days postpartum and manifests itself in the form of feeding difficulties, sucking or swallowing difficulties, and excessive crying in the newborn. Localised tetanus manifests mainly as muscle contractions at the site of injury. Cephalic tetanus manifests as cerebral nerve paralysis 1 to 2 days after infection.

### 3.4. Diagnosis and differential diagnosis

The “spatula” test, which involves gently touching the posterior pharyngeal wall with a tongue depressor or a soft instrument, shows reflexive spasms in tetanus patients and nausea reflexes in the normal population, and has a sensitivity of 94% and a specificity of 100% for the clinical diagnosis of tetanus.^[6]^ As the clinical manifestations of tetanus are more specific, typical symptoms, smear microscopy of wound secretion specimen, anaerobic culture, positive polymerase chain reaction of tetanus bacillus, and the “spatula” test can be used to assist in the diagnosis.

Tetanus is often misdiagnosed as cerebral infarction. Tetanus patients tend to be middle-aged or elderly, while cerebral infarction patients are mainly concentrated between the ages of 45 to 70 years old, and the 2 are similar in age characteristics. Cerebral infarction presents with symptoms similar to tetanus such as choking on water, difficulty in swallowing and abnormal muscle tone. In addition when patients present with tetanus symptoms and positive cranial MRI/CT, even if it is only old cerebral infarction or lacunar cerebral infarction, due to the similarity of the symptoms of tetanus and cerebral infarction,^[7]^ it is easy to be misdiagnosed as cerebral infarction by clinically inexperienced doctors,^[8]^ therefore cerebral infarction is often prioritized in clinical diagnosis and treatment rather than tetanus.^[9]^

Temporomandibular joint lesions can cause restricted movement of the jaw joints and pain in the masticatory muscles, resulting in restricted mouth opening and weak chewing, which can be easily confused with tetanus, and patients of such patients prefer to consult dental hospitals, whereas dentists often lack experience in the diagnosis and treatment of tetanus, which can easily lead to misdiagnosis.^[10]^

### 3.5. Tetanus treatment

Tetanus prevention relies primarily on antibodies acquired through active or passive immunization. Active immunization refers to the prophylactic administration of tetanus toxoid-containing vaccines to individuals at risk of infection prior to exposure to the tetanus bacillus (usually before trauma). The main tetanus toxoid-containing vaccines (TTCVs) currently in use globally are the tetanus vaccine, combined pertussis–diphtheria–tetanus (DTP) vaccine, combined diphtheria–tetanus (DTP) vaccine, and multiple vaccines, such as the combined pertussis–diphtheria–tetanus (PDT), inactivated polio-Haemophilus influenzae type b (Hib) vaccine and the combined pertussis–diphtheria–tetanus (PPT), and Hib (Hib) vaccine.

Passive immunological agents tetanus antitoxin and tetanus human immunoglobulin are effective in preventing post-traumatic tetanus. Tetanus immunoglobulin is derived from highly active antibody human plasma, free of preservatives and antibiotics, with higher stability to heat and cold, and a lower incidence of adverse reactions, making it more valuable for clinical application. Tetanus antitoxin (TAT) is not extracted from living organisms and is highly allergenic to the human body, and even non-allergic people may have reactions such as rash, nausea, and itchy skin when receiving this vaccine.^[11,12]^ Therefore, 3000 to 6000 IU multipoint intramuscular injections are clinically preferred to neutralize the toxin outside the CNS in the body, and if intramuscular tetanus immunoglobulin is not possible, TAT can be chosen to reduce morbidity and mortality.^[13]^

Antibiotics inhibit the proliferation of tetanus bacilli in wounds, and the recommended first-line agents are metronidazole and penicillin.^[14]^ Penicillin dosage is 80 to 1,000,000 U intramuscularly, once in 4 to 6 hours, or 200 to 1000 U intravenously, 2 to 4 times a day, however, penicillin has a noncompetitive, voltage-dependent inhibitory effect on γ-aminobutyric acid type A receptors, which attenuates the postsynaptic inhibitory response,^[15]^ and the use of high doses of penicillin may result in convulsions, myoclonus, coma, and severe psychiatric symptoms, which are called penicillin encephalopathy. The dosage of metronidazole is 2.5 g/d, orally or intravenously, 3 to 4 times a day, and the course of treatment is usually 7 to 10 days.^[16]^ Metronidazole not only has low tissue penetration, but also possesses γ-aminobutyric acid antagonist activity, which enhances the effect of tetanus spasmodic toxin.^[17]^ Therefore, some researchers believe that metronidazole is superior to penicillin and that metronidazole may be a better choice.^[18]^

### 3.6. Early proactive prevention for older persons

China eliminated neonatal tetanus in 2012 as a result of increased rates of hospital births, and the DTP vaccine was introduced in China in the 1960s. Therefore, people born before the 1960s (over 65 years of age) are in the immunization gap. However, since post-traumatic tetanus is not yet a legally reported infectious disease in China, resulting in a lack of accurate data on the incidence and disease burden of post-traumatic tetanus in the country, we believe that the patient may not have been actively immunized, thus providing a prerequisite for tetanus bacillus infection. Passive immunological agents have a short duration of protection, do not provide lasting protection for people with a high prevalence of trauma, and are associated with problems such as the risk of sensitization; TAT has been phased out in foreign countries, whereas tens of millions of TATs are used annually in China, presenting a potential healthcare safety issue.^[4]^ Therefore, the World Health Organization and countries such as the United States have recommended active immunization strategies for tetanus in high-risk populations, recommending an appropriate booster dose of TTCVs for the elderly, and the United States Advisory Committee on Immunization Practices has recommended that adults can be boosted with a dose of DPT vaccine every 10 years to maintain adequate immunoprotective antibodies.^[19]^ Most European countries recommend a regular booster dose of tetanus-containing vaccine every 10 to 20 years in adulthood.^[20]^ The UK guidelines for the management of tetanus recommend that if 5 doses of TTCVs are received before adulthood, the full immunization programme is considered complete, and routine boosters in adulthood have not yet been recommended for those who have completed their primary immunization. Compared with developed countries such as Europe and the United States, which have formed a more systematic active immunization strategy against tetanus in the adult population, most developing countries or regions have not made clear recommendations for tetanus vaccination in adulthood, nor have they introduced an immunization strategy for the routine vaccination of adults against tetanus, and have mostly adopted passive immunization to provide protection for the population after tetanus exposure.^[21]^ Therefore, in order to reduce the risk of tetanus in the elderly, and taking into account the recommendations of other countries, it is recommended that elderly people over 65 years of age should receive 3 doses of tetanus vaccine on a voluntary basis, and at least 1 dose should be given in consideration of the adherence of the elderly to vaccination.

### 3.7. Early proactive prevention for susceptible populations

Humans have no natural specific antibodies to the tetanus bacillus, and the risk of trauma (or burns) is significantly higher than in the general population for military personnel, police officers, firefighters, field engineering workers, construction workers, field workers in agriculture, forestry, fisheries and other fields, athletes, field sports enthusiasts and travelers. A health economics study in Colombia showed that booster immunization with tetanus vaccine every 10 years is more cost-effective than no booster immunization.^[22]^ Most countries and regions in Europe and the United States recommend booster immunization with tetanus vaccine every 10 years.^[20]^ Therefore, we suggest that priority attention should be given to new students in the military (including military academies), police academies and sports schools, or new recruits, construction workers, etc, who may be considered to receive a booster dose of tetanus vaccine, and thereafter the number of booster doses at 10-year intervals should be considered according to their occupations and exposure risks.

### 3.8. Ensure passive immunization of patients

Tetanus bacillus is an anaerobic bacterium. If the wound is narrow and deep, a closed environment will be formed, and the tetanus bacillus in the wound will release exotoxin continuously, which will be easy to multiply. In this case, the patient was admitted to the hospital and when the wound was debrided, a clay-like substance was found adhering to the distal end of the tendon, and we believed that the tetanus bacillus originated from the clay, and we “took for granted” that the patient had been passively immunized at the time of his admission to our hospital.

### 3.9. Early bacterial culture of fresh wounds

Infection is the main cause of recurrent symptoms in our patient, if the pathogenic bacteria that may cause infection can be speculated before infection occurs in open fracture, and then targeted use of sensitive antimicrobials, the rate of infection can be effectively reduced, and the preventive effect of antibiotics can be improved.90% of the pathogenic bacteria cultured after infection of open fracture are the same as the results of the initial wound bacterial culture, that is, it is considered that the bacterial culture prior to debridement has a presumptive value for the pathogenic bacteria that cause the the causative organisms responsible for the occurrence of infection in the wound has a presumptive value.^[23]^ The patient was treated surgically for an open fracture in a local hospital without bacteriological culture. Although bacteriological cultures were performed at the time of surgery in our hospital, the results did not suggest that the disease was a tetanus bacillus infection, which we believe may be due to the fact that the wound was not a fresh wound on the one hand, and on the other hand, tetanus bacillus requires a special culture medium for cultivation. Therefore, in the treatment of open wounds, bacteriological culture of fresh wounds in preoperative emergencies is valuable, and it is essential to carry out bacteriological culture to guide therapeutic use in order to prevent the occurrence of infections and target anti-infective therapy.

### 3.10. Open wounds are thoroughly debrided

Early and thorough debridement of open wounds can effectively remove the residual tetanus bacillus and its spores, reduce the release of toxins, shorten the course of the disease, reduce the morbidity and mortality rate, and is an important measure for the treatment of tetanus. Therefore, in the process of open wound diagnosis and treatment, the infected wounds and tissues should be thoroughly debrided, disinfected, and necrotic tissues should be removed, and any wounds where necrotic tissues can be found to remain in the wound memory and where drainage is not smooth should be debrided. Wounds that appear to have healed may have sinus tracts or submerged dead space and need to be carefully examined to remove necrotic and unhealthy tissue and open the wound for adequate drainage. Inadequate initial debridement of the patient creates “favorable” conditions for tetanus bacilli to flourish.

### 3.11. Ineffective nerve blocks indicate high nerve abnormalities

In this case, after the second postoperative continuous nerve block anesthesia, the symptoms of muscle twitching, spasm and rigidity of the right lower limb were slightly relieved but recurred. If the symptoms were caused by the nerves, the uncomfortable symptoms would have been relieved after the nerve block at that stage, but the patient relapsed once again, which indicated that the symptoms were not innervated by the nerves at that stage. Combined with the pathological mechanism of tetanus, we believe that in clinical diagnosis and treatment, if the symptoms are not relieved after nerve block at the site of injury, priority should be given to the abnormality of the high nerve, and patients will benefit from searching for the cause of the abnormality of the nerve, and the ineffectiveness of anesthesia of nerve block may be able to be used as a reference indicator of tetanus bacillus infection to assist in the diagnosis and treatment.

### 3.12. Vancomycin bone cement overlay for anti-infection

The patient had an open fracture and was treated with internal fixation under conditions of uncontrolled infection, and there was a lapse in treatment decision-making. Antibiotic-carrying bone cement has been widely used by scholars at home and abroad for the treatment of various wound infections and infectious diseases that are difficult to control.^[24]^ The antimicrobial activity of vancomycin mainly targets common causative organisms of bone infections, and the filling of vancomycin-containing bone cement cushions accurately and directly acts on the local area of injury, which ensures that the dead space as well as the area of poor blood flow can also have a high concentration of antibiotics.^[25]^ Through the slow release technology to control the slow release of drugs, to maintain the effective bacteriostatic concentration for a longer period of time, so that the drug action time to maintain months or even years, to reduce the pain of patients.^[26]^ Cemented drug-carrying cover can be locally administered without elimination through blood circulation and liver, reducing drug loss and enhancing bioavailability to achieve the purpose of improving the cure rate of infection. Local release of high concentrations of antibiotics kills bacteria that may remain after debridement, yet the local dose of vancomycin that enters the circulation is much lower than the systemically administered dose, resulting in milder adverse effects.^[27]^ The patient had a skin defect on the dorsum of the right foot, and after the infection was controlled, the patient’s suffering due to *Mycobacterium tetani* infection was taken into account, combined with the fact that the surgical site was susceptible to pathogens such as bacteria and viruses after flap grafting, which led to implant failure. Partial or total necrosis of the skin flap, scar formation and hyperpigmentation occur due to poor blood circulation, local pressure, ischemia and hypoxia. The location where the skin is removed may be unfavorable with pain, bleeding, infection and poor healing. We ultimately use vancomycin bone cement to cover the trauma.

### 3.13. Closed negative pressure drainage of the wound promotes anaerobic bacteria colonization

It was found that the lower limb skin defects in a single continuous negative pressure treatment within 11 days of negative pressure closed drainage does not achieve complete removal of traumatic bacteria, with the advancement of time, the bacterial species, the number of bacteria increased, the bacterial positivity rate overall tends to increase, the bacteria are mainly gram-negative bacteria, anaerobic bacteria and fungal growth in the negative pressure environment.^[28]^ And this patient underwent one traumatic closed negative pressure drainage after trauma and 2 traumatic closed negative pressure drainage in our hospital, which undoubtedly provided conditions for the propagation of tetanus bacilli. Therefore, the use of closed negative pressure drainage of the wound is avoided when there is clinical suspicion that the infecting bacteria are anaerobes.

Through the diagnosis and treatment of this patient, we believe that in the diagnosis and treatment of patients with open wounds, regardless of the size of the wounds, we should first ask the patients whether they had active immunity in the past, and if not, we should give them passive immunity in time. Typical tetanus symptoms are characterized by muscle tonus, spasms and autonomic dysfunction. In the course of clinical diagnosis and treatment, tetanus infection should be considered as the first step in patients with open wounds who have muscle tonus, spasms and autonomic dysfunction, and who have a positive “squeegee” test. Bacteriological cultures of fresh wounds in patients with open wounds allow an early diagnosis. Ineffective neural tissue anesthesia can be used as a secondary diagnostic indicator of tetanus infection. Avoid the use of closed negative pressure drainage of the wound when there is clinical suspicion that the infecting bacteria are anaerobic. When the patient has a skin defect, vancomycin bone cement coverage is an option after thorough debridement. Tetanus diagnosis and treatment is more preventive than curative, and early active immunization will allow people to benefit.

## Acknowledgments

Thanks to the editorial board, the reviewers, and all the authors for their dedication.

## Author contributions

**Conceptualization:** Xiaoqiang Wang, Shihong Xu.

**Data curation:** Xiaoqiang Wang, Shaobo Jing, Kai Li, Zhaoyang Jiang.

**Software:** Sixuan Chen.

**Supervision:** Jun Zhao, Xingwen Xie, Xingsheng Wang.

**Writing – original draft:** Xiaoqiang Wang.

**Writing – review & editing:** Xiaoqiang Wang, Xingzhang Yao.
